# Diagnostic accuracy of monoclonal antibody based serum immunoglobulin free light chain immunoassays in myeloma cast nephropathy

**DOI:** 10.1186/1472-6890-12-12

**Published:** 2012-08-09

**Authors:** Colin A Hutchison, Paul Cockwell, Mark Cook

**Affiliations:** 1 Renal Institute of Birmingham, University Hospital and University of Birmingham, Birmingham, UK; 2 Department of Haematology, University Hospital Birmingham, Birmingham, UK

**Keywords:** Acute kidney injury, Multiple myeloma, Free light chain, Cast nephropathy, Serum immunoassays

## Abstract

**Background:**

The development of serum immunoassays for the measurement of immunoglobulin free light chains has led to a paradigm shift in the diagnosis, assessment and monitoring of patients with plasma cell dyscrasias. The impact of these immunoassays which employ polyclonal antibodies was most notable for those patients who were previously classified as non-secretory multiple myeloma. Recently new monoclonal antibody based assays have become available. The purpose of this study was to compare the diagnostic sensitivity of these new assays with those already in clinical practice.

**Methods:**

Sera from 30 patients who present with severe acute kidney injury and multiple myeloma were identified for analysis. A head to head comparison of the two commercially available free light chains assays was then undertaken to determine if their diagnostic sensitivity and specificity were comparable.

**Results:**

In this first assessment of the utility of these new assays, we found that one of 17 patients with a lambda monoclonal free light chain resulting in acute kidney injury were not identified and a further 12% of patients were wrongly classified as having levels below those associated with disease specific acute kidney injury.

**Conclusion:**

These results suggest that caution should be applied to the use of new free light chain assays in the assessment of patients with a monoclonal gammopathy.

## Background

The development of polyclonal nephelometric immunoassays for the measurement of free immunoglobulin light chains (FLC) in the serum has changed screening algorithms, classifications and the monitoring of a number of plasma cell dyscrasias [1-3]. These assays, which utilise latex-conjugated polyclonal antisera, when combined with serum protein electrophoresis provide a sensitive screening tool for plasma cell dyscrasias. Together they identify all cases of multiple myeloma, 96% of AL-amyloidosis and 85% of monoclonal gammopathy of undetermined significance (MGUS) [4]. These FLC assays have a particular role for the diagnosis and monitoring of light chain only multiple myeloma [1], but in addition their sensitivity has lead to the development of a new classification of response of multiple myeloma: stringent complete response (when the FLC ratio has normalised, in addition to standard parameters). Furthermore the assessment of serum FLC provides an additional tool for the risk stratification of MGUS [5] and asymptomatic myeloma [6] and has allowed the description of a new class of FLC only MGUS [7].

Monoclonal serum FLC exhibit different inter-patient [8] and intra-patient [9] physio-chemical properties. This may reflect the genetic [10] and biological diversity of the FLC’s and contribute to one of the most significant complications of monoclonal FLC, acute kidney injury (AKI) secondary to cast nephropathy; when severe this has a profound impact on morbidity and mortality and reduces quality of life. The rapid diagnosis of AKI due to myeloma cast nephropathy facilitated by monoclonal FLC, allows the rapid initiation of disease specific treatment [11]. Recent work has demonstrated that when treatments are targeted to provide a rapid reduction in circulating concentrations of monoclonal FLC renal recovery occurs in the majority of patients [12-15].

Recently, new immunoassays which use monoclonal antibodies against FLC have become commercially available. The purpose of this study was to compare the diagnostic sensitivity of these new monoclonal assays with the established polyclonal FLC assays in the context of individuals presenting with new severe AKI secondary to multiple myeloma where there is a clear need for a rapid diagnosis.

## Results and discussion

**Figure 1:**
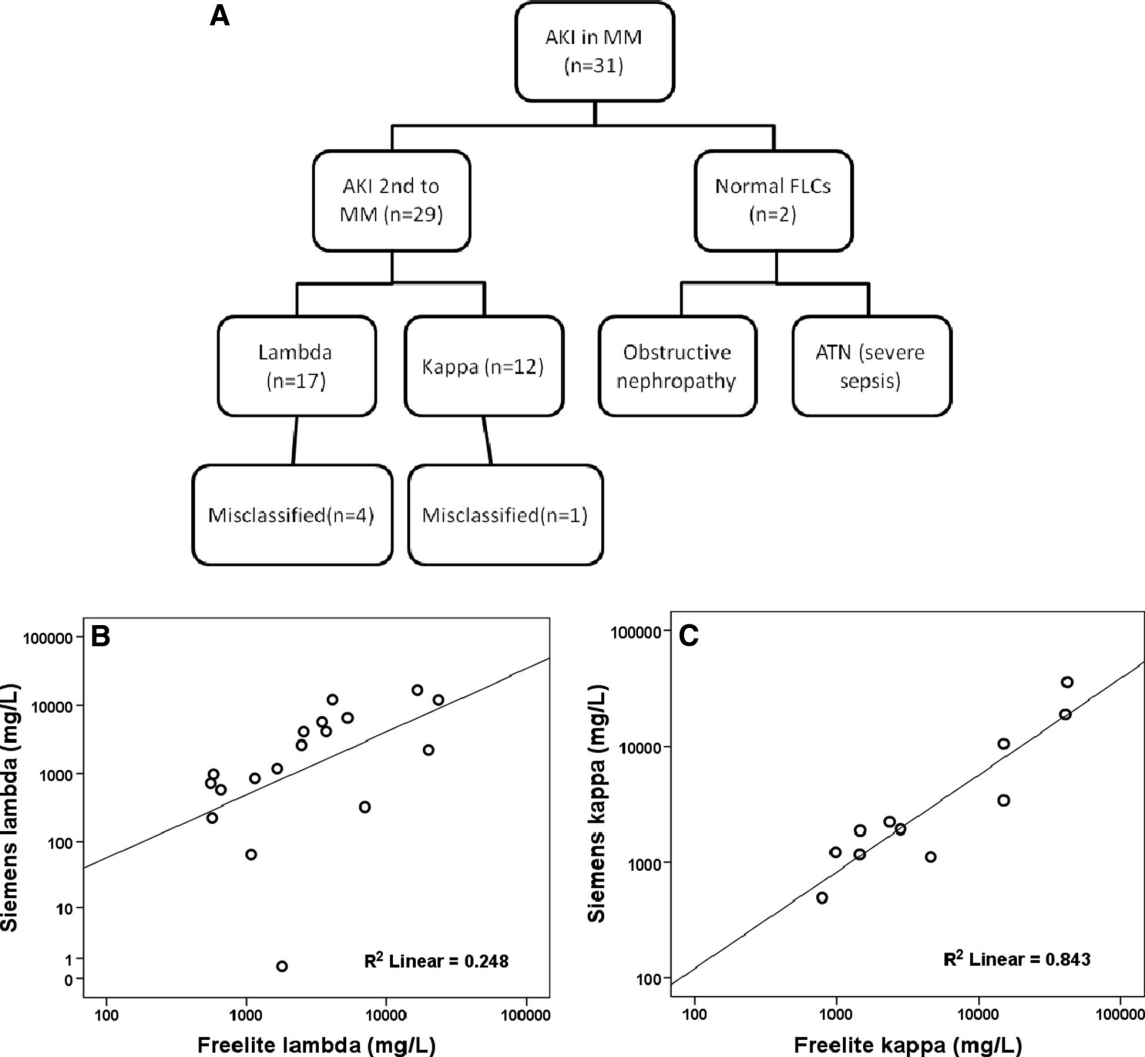
**Comparison of established (Freelite) and novel (Siemens) serum free light chain assays in patients with multiple myeloma and acute kidney injury.** The new serum FLC assays did not identify nephrotoxic levels of monoclonal FLCs in 18% of patients studied (**A**), largely this can be explained by the poor performance of the lambda assay (**B**). In comparison the correlation of the kappa assays was reasonable (**C**).

The purpose of this study was to compare the diagnostic sensitivity of the two commercially available immunoassays for the identification of monoclonal FLC, in patients with severe AKI and multiple myeloma. Similar reference range comparisons between the two assays had previously been reported [16], therefore for these assays to be used interchangeably it is appropriate to utilise clinical cut-offs identified by the predicate assays. Sera from 30 patients with severe AKI and multiple myeloma were available for analysis. Patients had a median age of 68.5 years and 70% were male. Seventeen patients had a monoclonal λ FLC, 11 patients had a monoclonal κ FLC and two patients had no demonstrable monoclonal FLC (Table). The renal diagnoses in the two patients without a monoclonal FLC were acute tubular necrosis in the context of severe infection and obstructive nephropathy, respectively. In the remaining 28 patients the cause of the AKI was attributed to multiple myeloma and a nephrotoxic FLC (Figure 1A).

The established FLC assays, which utilise polyclonal antibodies, have high rates of diagnostic sensitivity in patients with myeloma kidney [11], multiple myeloma, AL-amyloidosis and MGUS [17]. In this first clinical comparison of the established assays with the new assays, which use monoclonal antibodies, we found one of the seventeen patients (6%) with a monoclonal λ FLC was miss-classified by the assay (Tables 1 and 2). The patient and 7, had 1810 mg/L of λ FLC with the historic assays and 1 mg/L λ FLC / 4 mg/L κ FLC by the new assay incorrectly indicating a kappa clone, in accordance with manufacturers instructions antigen excess checks were not performed. All patients with a monoclonal κ FLC were identified with the new assay. The missed case of λ FLC monoclonality indicates the limitation of using monoclonal antibodies, as they have a limited number of epitopes which they can identify; the myriad structural variations in monoclonal FLC means that there will be cases where a monoclonal antibody based assay will not detect FLC clonality; this case is a practical demonstration of this principle.

**Table 1:** Summary for comparison of diagnostic accuracies between the two free light chain assays in patients with multiple myeloma and acute kidney injury

			**Siemens**		**% misclassified by Siemens Test**
			**Yes**	**No**	
**LCMM**	**Freelite**	**Yes**	7	1	12.5%
		**No**	0	0	
**IIMM**		**Yes**	17	4	19%
		**No**	0	0	

The table above shows the number of patients that tested positive or negative with the Freelite and Siemens FLC assays. The Siemens FLC assay misclassified 5/28 patients with monoclonal FLC >500 mg/L. LCMM – light chain multiple myeloma, IIMM – intact immunoglobulin multiple myeloma.

**Table 2:** Demographics of the 5 patients missed by the Siemens FLC assay

**Patient**	**MM type**	**Freelite**			**Siemens**			Missed FLC*	Misclassified FLC as <500 mg/L**
		**κ**	**λ**	**Ratio**	**κ**	**λ**	**Ratio**		
**2**	Free λ	2	7010	0.0003	1	322	0.003	No	Yes
**7**	IgA λ	8	1810	0.0044	4	1	8.058	Yes	Yes
**10**	IgG λ	8	1080	0.0078	9	64	0.134	No	Yes
**13**	IgG λ	9	572	0.0162	9	225	0.041	No	Yes
**23**	IgG κ	796	6	125.7	493	17	29.6	No	Yes

*Patients were classified as missed FLC if the new assays did not identify a monoclonal FLC.

**Patients were classified as misclassified if their monoclonal FLC level was reported as less than the nephrotoxic level of 500 mg/L (5 of 28 patients).

The correlation coefficient of the two FLC assays was less good for patients with monoclonal λ FLC (Figure 1B) than for patients with monoclonal κ FLC (Figure 1C): r = 0.617, p < 0.003 and r = 0.843, p = 0.001 respectively. The correlation of the FLC ratios between the two assays was 0.894, P < 0.01. The shortfalls in correlations of the absolute values of FLC are relevant for both the diagnosis and monitoring of patients. First, five of the 28 patients (18%) with AKI secondary to multiple myeloma were misclassified by the new assays as having FLC levels below the nephrotoxic threshold of 500 mg/L, four of these patients had a monoclonal λ FLC and one had a monoclonal κ FLC. Second, disease response as defined by the reduction in serum FLC levels is increasing in importance in all patients with multiple myeloma, but has particularly relevance in those with AKI where an early reduction in monoclonal FLC both guides treatment and suggests outcome [12-15]. It is imperative therefore that the established ‘risk’ and ‘response’ levels of the original FLC assays cannot be used for the new FLC assays. Rather levels specific for the new assays should now be identified. Clinicians will then need to be made aware of these new levels and informed which assay their laboratory is using.

The principal limitation of this work is the small population size. Further work should now be undertaken to assess these new monoclonal assays in larger populations and specific disease groups such as AL-amyloid, where the potentially inferior performance of the new λ assay may be of particular importance. In addition, the correlations between these assays are not robust enough to apply the current definitions of myeloma response based on FLC monoclonality from the new assays.

## Conclusion

This study highlights the importance of large comparative studies of new immunoassays before they are used in clinical practice. This provisional work demonstrated that the new monoclonal N-latex assay for FLC measurement did not robustly replicate the results of the established polyclonal FLC assays. Further work is now required before these new assays can be adopted into clinical practice.

## Methods

This study was undertaken as a service evaluation at the University Hospital Birmingham. All data analysis was coded and anonymised. Sera from patients, who presented with new dialysis dependent renal failure, to the renal unit at the University Hospital Birmingham, were screened for inclusion in a trial assessing the management of severe renal failure in multiple myeloma (COREC 05/Q2706/107, South Birmingham Research Ethics Committee), results of which have been reported previously [14,18,19].

### Study populations

Dialysis-dependent renal failure was defined as an estimated GFR of <15mls/min/1.73 m^2^, as calculated by the abbreviated MDRD equation and a clinical or metabolic requirement for dialysis as identified by the reviewing nephrologist [20]. Attribution of the cause of renal failure to multiple myeloma was based on renal histology or in cases where a renal biopsy was contraindicated when all other potential causes were excluded. The clinical diagnosis of multiple myeloma was made by a consultant haematologist in accordance with international diagnostic criteria [21].

### Laboratory and statistical analysis

Serum samples were stored at −80°C until thawed for the current study; previous work has demonstrated the stability of FLC concentrations in urine samples over many years [22] and apparent stability in serum samples [17]. Serum κ and λ FLC concentrations were measured by nephelometry, on a Dade-Behring BN™II Analyser, using particle-enhanced, high-specificity, homogeneous immunoassays (Freelite™, The Binding Site Group Ltd, Birmingham, UK [23] and N-Latex FLC, Siemens Healthcare Diagnostic Products GmBh, Germany). FLC results were compared with the published reference range for the FLC ratio in patients with renal failure reference range (0.37-3.17) [24].

Data were analysed using SPSS 17.0 for Windows. Spearman’s correlation coefficient was used to assess the correlation of results between the two assays and Chi square test to assess the classification of the patients by the two assays.

## Competing interests

CH and PC have received research funding from the Binding Site, CH is a consultant to the Binding Site.

## Authors’ contributions

All authors were involved in the study design and manuscript preparation. CH undertook the data analysis. All authors read and approved the final manuscript.

## Pre-publication history

The pre-publication history for this paper can be accessed here:

http://www.biomedcentral.com/1472-6890/12/12/prepub
